# PD52 - Role of atypical pathogens mycoplasma pneumoniae and chlamydia pneumoniae in childhood asthma exacerbations

**DOI:** 10.1186/2045-7022-4-S1-P52

**Published:** 2014-02-28

**Authors:** Dorina Savoschin, Ala Cojocaru, Liubov Vasilos, Olga Cirstea, Alexandra Guscov, Tatiana Culesin, Lilia Chiosea

**Affiliations:** 1 Institute for Maternal and Child Healthcare, Chisinau, Moldova Republic

## Background

The role of *Mycoplasma pneumoniae* and *Chlamydia pneumoniae* infection in acute asthma is determined by the ability of these pathogens to initiate the development of bronchial obstruction and to increase the frequency and severity of the disease exacerbations. Studies that have been previously published showed contradictory results, thus further research on this subject need to be performed.

Our study **aimed** to reveal immunological peculiarities in patient with asthma triggered by atypical pathogens in order to improve diagnosis and management of this category of patients.

## Materials and methods

The study included 54 children aged from 1 to 18 years, hospitalized with asthma exacerbation. In all cases immunological essays were performed in order to assess specific antibodies (IgA/ IgM/ IgG to *Mycoplasma pneumoniae* and *Chlamydia pneumoniae*; total serum IgE levels examined using ELISA method.

## Results

Serological markers for atypical pathogens were identified in 32 (59%) children investigated. Acute *Mycoplasma pneumoniae* infection was estimated in 13% of cases, chronic infection with *Mycoplasma pneumoniae* was diagnosed in 26% of children. *Chlamydia pneumoniae* caused acute infection in 20.4% of those investigated. Also, total serum IgE levels in children with asthma and acute infection was 1.5-fold higher than in the serologically-negative group (916.0±236.0 IU/ml vs. 647.9±104.6 IU/ml, respectively, p>0.05). Likewise, a significant correlation of the total serum IgE levels with IgG anti*-Mycoplasma pneumoniae* antibodies was identified (r=0,58; p<0,01). This indicates that *Mycoplasma pneumoniae* infection may be involved in mechanisms of allergic sensitization, *hyper-IgE production* and asthma pathogenesis in children.

## Conclusions

Immunological diagnosis of infections with atypical pathogens associated with asthma exacerbations may be helpful in improving the complex management of children with asthma. It offers the possibility to use the ethiotropic antibacterial therapy that may have a positive impact on the disease severity and the level of asthma control.

